# Immune signaling and function in neurodegeneration

**DOI:** 10.1172/JCI199850

**Published:** 2026-04-15

**Authors:** Yvonne L. Latour, Dorian B. McGavern

**Affiliations:** Viral Immunology and Intravital Imaging Section, National Institute of Neurological Disorders and Stroke (NINDS), National Institutes of Health (NIH), Bethesda, Maryland, USA.

## Abstract

Neurodegenerative diseases arise from interactions among pathogenic proteins, immune responses, and diverse environmental or age-related stressors that disrupt CNS homeostasis. CNS resident microglia detect self-derived danger signals through pattern recognition receptors, and their activation can promote clearance of aberrant proteins, including amyloid-β, tau, α-synuclein, and TAR DNA-binding protein 43. However, microglial activation may also drive maladaptive states that amplify neuroinflammation. Microglial transitions are further shaped by receptor-mediated signaling and antigen presentation pathways that integrate environmental cues with functional responses. Adaptive immune cells contribute additional layers of regulation, with CD8^+^ and CD4^+^ T cells exerting neuroprotective or neurotoxic effects depending on disease context, activation state, and antigen specificity. The identification of granzyme K–expressing CD8^+^ T cells in several neurodegenerative conditions highlights the growing recognition that distinct T cell subsets may have specialized roles in disease. Aging, repetitive head injury, and viral infection further alter microglial phenotypes, weaken barrier integrity, promote T cell recruitment, and prime the CNS for chronic inflammation. In this review, we synthesize current knowledge of innate and adaptive immune mechanisms in neurodegeneration, examine how external factors influence these responses, and consider how these insights may guide future therapeutic strategies.

## Introduction

Neurodegenerative diseases remain a major clinical concern, affecting millions of individuals worldwide. Although a wide range of disorders fall under the neurodegenerative disease umbrella, many share hallmark features, including neuronal cell death, cognitive dysfunction, and aberrant protein accumulation, which have been reviewed elsewhere (1). Our understanding of the pathophysiology of Alzheimer’s disease (AD), Parkinson’s disease (PD), Huntington’s disease (HD), Lewy body dementia (LBD), amyotrophic lateral sclerosis (ALS), and frontotemporal dementia (FTD) has expanded beyond the classical association of neuronal loss and immune reactivity localized to regions of protein aggregation. This shift has been driven by the availability of new research tools and approaches (2–7) that have revealed the immune system not only as a key driver of neurodegenerative disease progression but also as a contributor to disease onset through maladaptive cellular activation and signaling (2–4, 8).

Under steady-state conditions, the CNS parenchyma relies on resident immune cells to maintain the blood–brain barrier (BBB), survey the local environment, and respond to cellular damage, pathogens, or dysfunctional cells. Neurons activate intrinsic signaling pathways in response to cellular dysfunction, while astrocytes and microglia are rapidly engaged to mitigate danger signals and, when necessary, recruit peripheral immune cells. During neurodegeneration, however, these responses can exacerbate aberrant protein deposition, and the balance of beneficial versus detrimental immune involvement remains incompletely understood. In this review, we explore key signaling pathways, cellular players, and immune responses that are shared across multiple neurodegenerative diseases, and we discuss how additional factors such as aging, infection, and repetitive injury may influence disease progression within the CNS.

## Innate immune signaling

**Figure 1:**
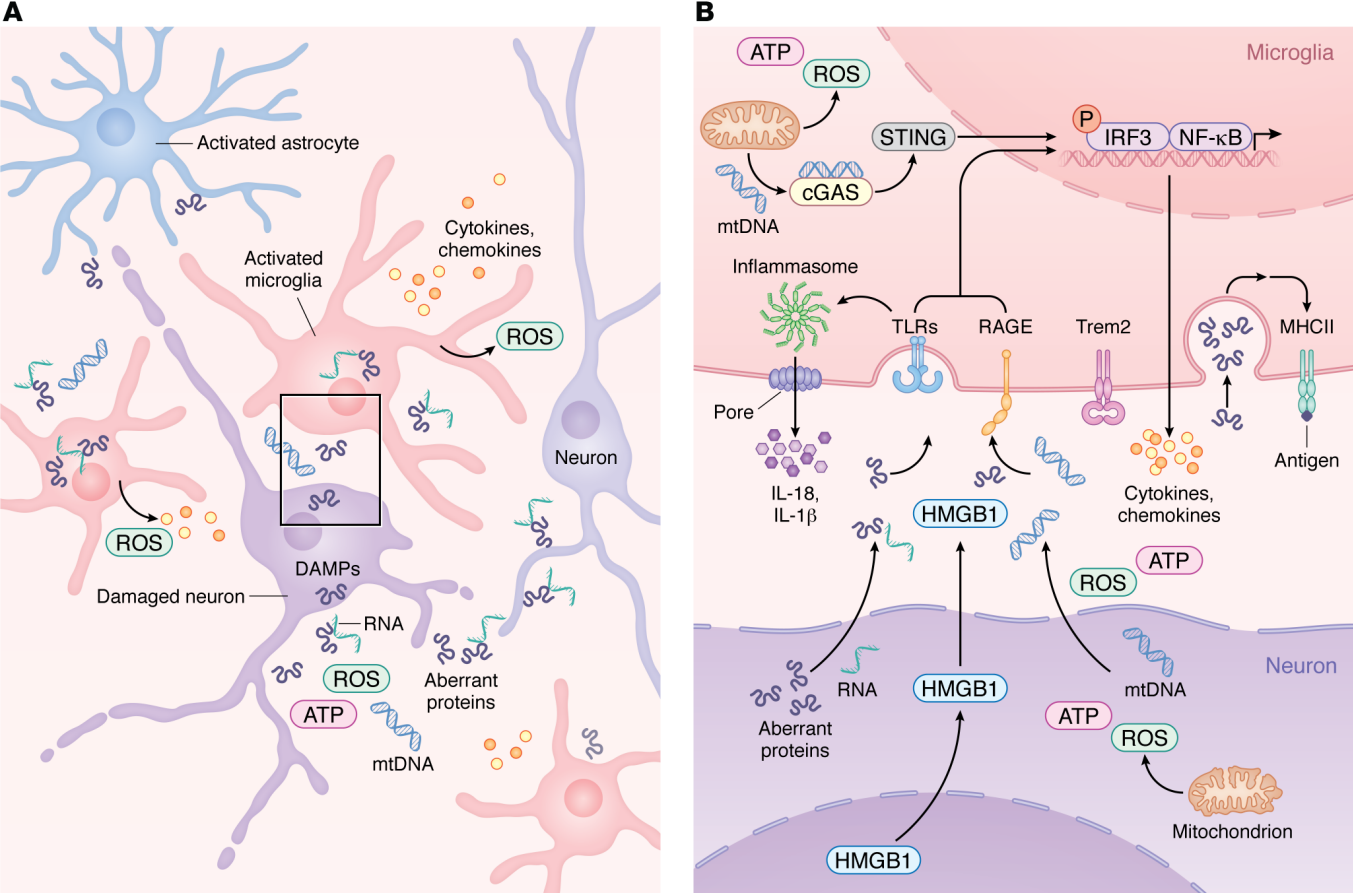
Innate immune activation and microglial transition during neurodegeneration. (**A**) During neurodegeneration, distressed neurons release DAMPs such as mtDNA, ROS, HMGB1, aberrant proteins, and pTau–bound RNA. These signals activate resident microglia and astrocytes, which then recruit peripheral immune cells. As shown in more detail in **B**, activated microglia upregulate PRRs, including TLRs, RAGE, and cGAS, enabling detection of neuron-derived DAMPs. Engagement of PRRs induces proinflammatory gene expression programs and activates inflammasome pathways that promote release of chemokines and cytokines into the CNS parenchyma. As microglia transition from homeostatic to reactive states in a TREM2-dependent manner, damage-associated microglia increase MHC-II expression to support antigen presentation and coordination of adaptive immune responses that attempt to limit neurodegeneration.

The innate immune response has evolved mechanisms to detect both externally derived stimuli, known as pathogen-associated molecular patterns (PAMPs), and endogenous molecules released from stressed or dying cells, referred to as damage-associated molecular patterns (DAMPs) (Figure 1A). These signals are recognized through pattern recognition receptors (PRRs) (9–11). As the primary resident immune cells of the CNS, microglia express high levels of PRRs; however, evidence also supports PRR expression on other CNS cell types, including astrocytes, oligodendrocytes, endothelial cells, and neurons (12–16).

### TLRs.

The most well-studied family of PRRs are the TLRs, first identified and characterized for their ability to sense a variety of microbial components (Figure 1B) (17–19). TLRs are transmembrane proteins that contain a leucine-rich repeat (LRR) extracellular domain and a cytosolic Toll IL-1 receptor (TIR) domain responsible for downstream signaling (18). When TLRs bind PAMPs, the intracellular domain recruits the adaptor proteins myeloid differentiation factor 88 (MyD88) or Tir domain–containing adaptor inducing interferon β (TRIF). This process results in the nuclear translocation of transcription factors such as NF-κB, which initiates proinflammatory gene expression programs (18, 20). Later studies showed that TLR activation can also occur in response to DAMPs during sterile inflammation in the CNS (21).

As a primary hallmark of neurodegenerative diseases, aberrant proteins such as amyloid β (Aβ), phosphorylated tau (pTau), α synuclein (α-syn), and TAR DNA binding protein 43 (TDP-43) have been identified as major DAMPs that activate PRRs in the CNS (22, 23), and these proteins are sometimes complexed with nucleic acids like RNA (Figure 1B) (24–29). Plasma membrane–bound TLR2 and TLR4 are upregulated in the brains of patients with AD and in transgenic AD mouse models (30). Binding of Aβ to TLR2 and TLR4 increases expression of immunomodulatory cytokines, including IL-1β, IL-6, IL-10, IL-17, and TNFα, in microglia and astrocytes (31–34). Genetic ablation or pharmacologic blockade of TLR2 and TLR4 exacerbate cognitive decline and increase Aβ burden in the brains of 5xFAD and APP/PS1 mice by reducing microglial activation (32, 34–36). However, an appropriate balance must be maintained, since persistent activation of the proinflammatory response downstream of TLR4 can be detrimental and amplify neurotoxicity (37, 38). In addition, recognition of Aβ by TLR4 expressed on neurons induces apoptosis and contributes to neurodegeneration (16). Similar mechanisms have been observed in other neurodegenerative disease models. Extracellular pathogenic α-syn, superoxide dismutase 1 (SOD1), and tau released by damaged neurons activate TLR2 and TLR4 and promote microglial reactivity in vitro and in vivo in models of PD, ALS, and tauopathy (39–45).

### NLRs.

LRR-containing NLRs form a family of cytosolic PRRs composed of a pyrin domain that receives activation signals, an adaptor protein, and a caspase that is recruited after activation (46). The most relevant member in neurodegeneration is the NLRP3 inflammasome (Figure 1B), which includes the adaptor protein apoptosis-associated speck-like protein containing a caspase recruitment domain (ASC) and caspase-1 (42, 47). Canonical NLRP3 inflammasome activation is a multistep process. It begins with priming through TLR signaling, which increases transcription of NLRP3, pro-IL-1β, pro-IL-18, and the pore-forming protein Gasdermin D (GSDMD) (48, 49). A second signal, usually internalized PAMPs or DAMPs, activates the inflammasome and promotes maturation of caspase-1. Activated caspase-1 then cleaves pro-IL-1β, pro-IL-18, and GSDMD into their active forms for release (50).

Inflammasome activation in connection with AD was first described in 2008 by Halle et al. (51). Aβ fibrils induced IL-1β production by primary microglia in vitro, and NLRP3, caspase-1, and IL-1β were required to recruit microglia to areas of exogenous Aβ deposition (51). Subsequent studies showed that Aβ oligomers and fibrils, but not monomers, interact directly with NLRP3 to activate the inflammasome in a cell-free system and induce IL-1β release in culture (52). Stimulation of primary microglia ex vivo with Aβ oligomers and protofibrils did not impair cell viability, indicating that inflammasome activation in microglia does not result in pyroptosis, which is a common outcome of inflammasome activation (53). In humans with AD, cleaved caspase-1 is increased in tissue from the frontal cortex and hippocampus compared with control tissue (54). These findings were reproduced in APP/PS1 mice, and in that model, NLRP3 deficiency rescued memory impairment and reduced Aβ burden by increasing microglial Aβ clearance (54).

NLRP3 is also highly activated in microglia located in the substantia nigra of postmortem PD patient brains (55). Patients with PD exhibit elevated circulating IL-1β and caspase-1 in the blood and cerebrospinal fluid (CSF) (55, 56). Similar findings were observed in the preformed fibril and 6-OHDA mouse models of PD, in which fibrillar α-syn activated NLRP3, and oral administration of the NLRP3 inhibitor MCC950 reduced motor deficits, neuronal loss, and α-syn accumulation (55). Tau has also been shown to induce NLRP3 inflammasome activation in microglia (57–59). However, the role of inflammasome activity in tauopathy remains unclear, since conflicting results have been reported depending on the mouse model used. For example, loss of NLRP3 or ASC in the PS19 and Thy Tau22 models improved behavioral outcomes and reduced tau aggregation (57, 58, 60), whereas genetic deletion of *Nlrp3* did not alter disease progression or outcome in the P301S model (61).

### RAGE.

The receptor for advanced glycation end products (RAGE) is a multiligand cell surface receptor (Figure 1B). As a transmembrane protein, it contains an extracellular region that binds ligand, a membrane-anchoring region, and a cytosolic region responsible for intracellular signaling (62). RAGE exists in 2 forms; the membrane-bound form contains the full protein structure, while a soluble form contains only the extracellular domain and can neutralize circulating ligands to reduce cellular activation (63). Unlike the other PRR families described above, RAGE primarily recognizes DAMPs. These ligands include AGE, high mobility group box 1 (HMGB1), heat shock proteins (HSPs), S100 proteins, DNA, and in the setting of neurodegenerative disease, aberrant proteins (63). RAGE not only recognizes a wide range of ligands but also initiates diverse intracellular signaling pathways (64).

Studies in AD (65–67), ALS (68, 69), and PD (70–73) have consistently reported elevated RAGE expression on neurons, microglia, and astrocytes, suggesting a broad role for this receptor in neurodegenerative pathology. Functional studies reinforce this idea. Overexpression of RAGE in AD mouse models increased NF-κB translocation and accelerated cognitive impairment (74, 75), whereas RAGE deficiency mitigated amyloid pathology and reduced memory deficits (76). RAGE also contributes to Aβ dynamics at the BBB, as circulating Aβ can bind endothelial RAGE and be transported into the parenchyma, promoting both Aβ accumulation and production of proinflammatory cytokines (77). Consistent with this mechanism, inhibition of RAGE reduced Aβ levels in the Tg2576 model of AD, and an orally administered vaccine containing a RAGE and Aβ complex improved outcomes in APP/PS1 mice by inducing antibodies against both targets (78). Beyond Aβ, RAGE is activated by α-syn and induces production of TNF-α, IL-6, and IL-1β by microglia in culture (79), and RAGE deficiency is similarly beneficial in the SOD1 model of ALS, reducing gliosis and inflammatory marker expression (80). Collectively, these findings demonstrate that RAGE-mediated detection of aberrant proteins amplifies neuroinflammation across multiple neurodegenerative diseases, and a more complete understanding of the pathways downstream of RAGE may help identify therapeutic targets (81).

High mobility group box 1 (HMGB1) is a ubiquitously expressed protein that interacts with DNA to facilitate chromatin binding (82, 83). HMGB1 is detected by TLR2, TLR4, TLR9, and RAGE (84), and it is released by damaged neurons in culture where it promotes microglial activation (Figure 1B) (85, 86). In rats injected intrahippocampally with Aβ, HMGB1 accumulated around dying neurons associated with Aβ plaques. Coinjection of Aβ with exogenous HMGB1 enhanced neuronal loss and impaired microglial Aβ clearance (87). In a rat model of PD induced by 6-OHDA administration, treatment with a mAb targeting HMGB1 ameliorated motor deficits and reduced expression of IL-1β and IL-6 (88). Similarly, anti-HMGB1 mAb treatment delayed loss of grip strength in the SOD1 mouse model of ALS, although this benefit was observed only when treatment began before symptom onset (89).

### DNA sensors.

Cytosolic DNA sensors (CDSs) represent a class of receptors that detect pathogens or cellular damage. The recently identified cyclic GMP–AMP synthase (cGAS) detects cytosolic double-stranded DNA (dsDNA) and induces a type I interferon (IFN-I) response (Figure 1B) (90). Upon binding dsDNA, cGAS undergoes a conformational change and synthesizes cyclic GMP–AMP (cGAMP) from ATP and GTP (91). cGAMP then binds stimulator of IFN genes (STING), triggering phosphorylation and nuclear translocation of IFN regulatory factor 3 (IRF3) and induction of IFN-I expression (92, 93).

Unlike TLR2 and TLR4, TLR9 resides within late endosomes and detects intracellular DNA containing unmethylated cytosine–guanine (CpG) motifs (94). TLR9 activity is commonly assessed using type B CpG oligodeoxynucleotides (ODNs) (95–97). Activation of TLR9 was neuroprotective in the APP, TgSwDI, and 3xTg AD mouse models by enhancing immune responses and decreasing Aβ burden, although it also promoted tau phosphorylation (94–97). These findings highlight that DNA sensing within the endolysosomal system can modulate AD-related pathology and have prompted investigation into how cytosolic DNA sensing pathways contribute more broadly to neurodegeneration.

Among these mechanisms, the cGAS–STING pathway has emerged as a central modulator of neuroinflammation across multiple neurological disorders as reviewed extensively elsewhere (98–100). In the 5xFAD model, microglia interacting with amyloid plaques containing nucleic acids activated an IFN-1 signature that exacerbated Aβ pathology (101). Similar upregulation of IFN-stimulated genes (ISGs), consistent with activation of cytosolic DNA sensing, occurred in spinal cord astrocytes in the SOD1 model of ALS (102), demonstrating that these pathways are engaged across neurodegenerative contexts. This interpretation is further supported by detection of cGAS in human AD brain tissue, 5xFAD mice, P301S tauopathy mice, and AppNL-G-F/hTau double-knock-in mice (103–105). In these animal models, genetic ablation of cGAS or pharmacologic inhibition of STING reduced Aβ deposition, limited immune activation, and improved cognitive outcomes. Immunostaining revealed colocalization of STING with IRF3 in CD68-expressing cells, implicating microglial cGAS–STING signaling as a major contributor to disease-associated inflammation (103). Similar mechanisms extend to other proteinopathies: α-syn preformed fibrils induced DNA damage and activation of cGAS–STING in mixed glial cultures and in striatal microglia in a corresponding PD model, and loss of STING attenuated the IFN response and conferred neuroprotection (106).

Mitochondrial DNA (mtDNA) plays a pivotal role in activating cGAS–STING signaling during neurodegeneration. Under oxidative stress, mitochondria release ATP, ROS, and mtDNA into the cytosol (107), where the latter can engage cGAS (Figure 1B) (108). In P301S tauopathy mice, pathogenic tau triggered cytosolic release of mtDNA from microglia, initiating cGAS–STING signaling and upregulation of IFN-I and ISGs, whereas cGAS deficiency restored cognition and prevented neuronal loss (104). TDP-43 behaved similarly by entering mitochondria of iPSC-derived motor neurons and promoting mtDNA release, leading to cGAS–STING activation. Deleting or inhibiting STING in TDP-43–overexpressing mice prevented IFN-I induction and cortical degeneration (109). Beyond these models, heightened cGAS–STING signaling was also detected in mice carrying the 2 strongest genetic risk factors for AD, the APOE ε4 allele and the TREM2 R47H variant, and treatment with a cGAS inhibitor ameliorated tau-related pathology in that model (110). In parallel, recent work demonstrates that Aβ-induced oxidative stress drives cytosolic release of oxidized mtDNA fragments that adopt a Z conformation, which are sensed by Z-DNA–binding protein 1 (ZBP1) to activate RIPK1 and promote proinflammatory gene transcription in an AD mouse model (111). Collectively, these studies establish that activation of DNA-sensing pathways, whether endosomal or cytosolic, substantially contributes to neuroinflammation and neurodegenerative progression, providing a strong mechanistic rationale for therapeutic strategies targeting these innate immune axes.

### TREM2 signaling.

Microglia are the sentinels and first responders of the CNS parenchyma, and microgliosis, particularly in regions with aberrant protein accumulation, is a well-established hallmark of most neurodegenerative diseases (1, 112–115). A unique feature of microglia is their high expression of the triggering receptor expressed on myeloid cells 2 (TREM2) (Figure 1B). Early genetic studies in patients with AD identified the R47H and R62H SNP variants of *TREM2* as risk factors at a magnitude similar to that associated with possession of one *APOE* ε4 allele (116, 117). These discoveries positioned microglia as central players in neurodegenerative disease pathogenesis and accelerated efforts to clarify their functional roles. As research tools have advanced, our understanding of microglial heterogeneity and stimulus-dependent activation states has grown rapidly. A major conceptual advance in this area was the identification of disease-associated microglia (DAMs) in AD using single-cell sequencing approaches (118). During the transition from homeostatic to reactive states, microglia first downregulate markers such as P2RY12, CX3CR1, and TMEM119 (119), and then, in a TREM2-dependent manner, upregulate gene signatures associated with antigen presentation, lipid metabolism, and phagocytosis (120).

Mechanistic studies of TREM2 signaling demonstrate that this receptor activates a complex intracellular cascade that shapes microglial responsiveness during neurodegeneration. Although the R47H and R62H variants have been proposed to impair TREM2 function, animal studies manipulating TREM2 expression have produced mixed results (121, 122). Downstream of TREM2, activation of spleen tyrosine kinase (Syk) and engagement of the PI3K/AKT/GSK3β/mTOR pathway support microglial metabolic programs and phagocytic capacity (123). Reflecting the importance of this signaling axis, microglial Syk knockout in 5xFAD mice impaired Aβ uptake, increased Aβ burden, and worsened memory deficits by disrupting DAM-associated transcriptional and metabolic profiles (123, 124). Conversely, overexpression of human TREM2 in 5xFAD mice enhanced DAM gene expression, improved microglial phagocytic activity, and produced a protective phenotype (125). In line with these findings, TREM2 deficiency in 5xFAD mice increased Aβ burden, suppressed microglial activation, and exacerbated disease progression (126, 127).

Despite this overall pattern, the consequences of TREM2 loss vary across disease stage and neurodegenerative model. In the APP/PS1 model of AD, TREM2 deficiency delayed early amyloid plaque accumulation but ultimately exacerbated pathology at later stages (128, 129). Loss of TREM2 also impaired microglial clustering around plaques, a finding mirrored in postmortem AD brain tissue from individuals carrying R47H and R62H haplodeficient variants (128–130). Beyond AD, TREM2 signaling appears neuroprotective in mice expressing human TDP-43, in which TREM2-deficient microglia failed to phagocytose TDP-43 inclusions and remained in a homeostatic state (131). In tauopathy models, the effects are more complex. In the PS19 mouse line, TREM2 deficiency reduced microgliosis and attenuated microglial activation without altering pTau aggregation (132). However, a study using P301S mice argued that full *Trem2* knockout produces an artificially protective phenotype and that *Trem2* haplosufficiency, which more accurately reflects human haplodeficiency, is detrimental (133). Collectively, these studies establish TREM2 signaling as a central regulator of microglial activation and function across multiple neurodegenerative diseases and highlight the therapeutic potential of modulating DAM states (134).

### MHCII presentation.

As myeloid-derived immune cells, microglia also function as antigen-presenting cells capable of processing antigens and presenting them to T cells through MHCII (135). Immunostaining of the CNS in TE4 tauopathy mice carrying human *APOE4* showed microglia with elevated MHCII expression concentrated in tau-rich regions. Moreover, DAM-like CD11c^+^ microglia were frequently positioned adjacent to CD8^+^ T cells, consistent with potential cellular interactions. Similar patterns were observed in the parenchyma of APP/PS1 mice, where MHCII^+^ microglia were present near amyloid plaques, although they appeared more sparsely distributed (136). In the P301S model of tauopathy, a substantial population of CD11c^+^ MHCII^+^ microglia emerged in the spinal cord of symptomatic mice (137). MHCII expression is likewise increased in microglia in response to α-syn overexpression in vivo. When primary microglia were cultured with α-syn ex vivo, antigen processing was enhanced, and the microglia were able to induce activation and proliferation of CD4^+^ T cells. MHCII deficiency reduced microgliosis and prevented α-syn–induced neuronal death (138). Collectively, these studies demonstrate extensive coordination between microglia and the adaptive immune response in neurodegenerative disease and emphasize the importance of antigen presentation pathways in disease progression.

## T cell responses

**Figure 2:**
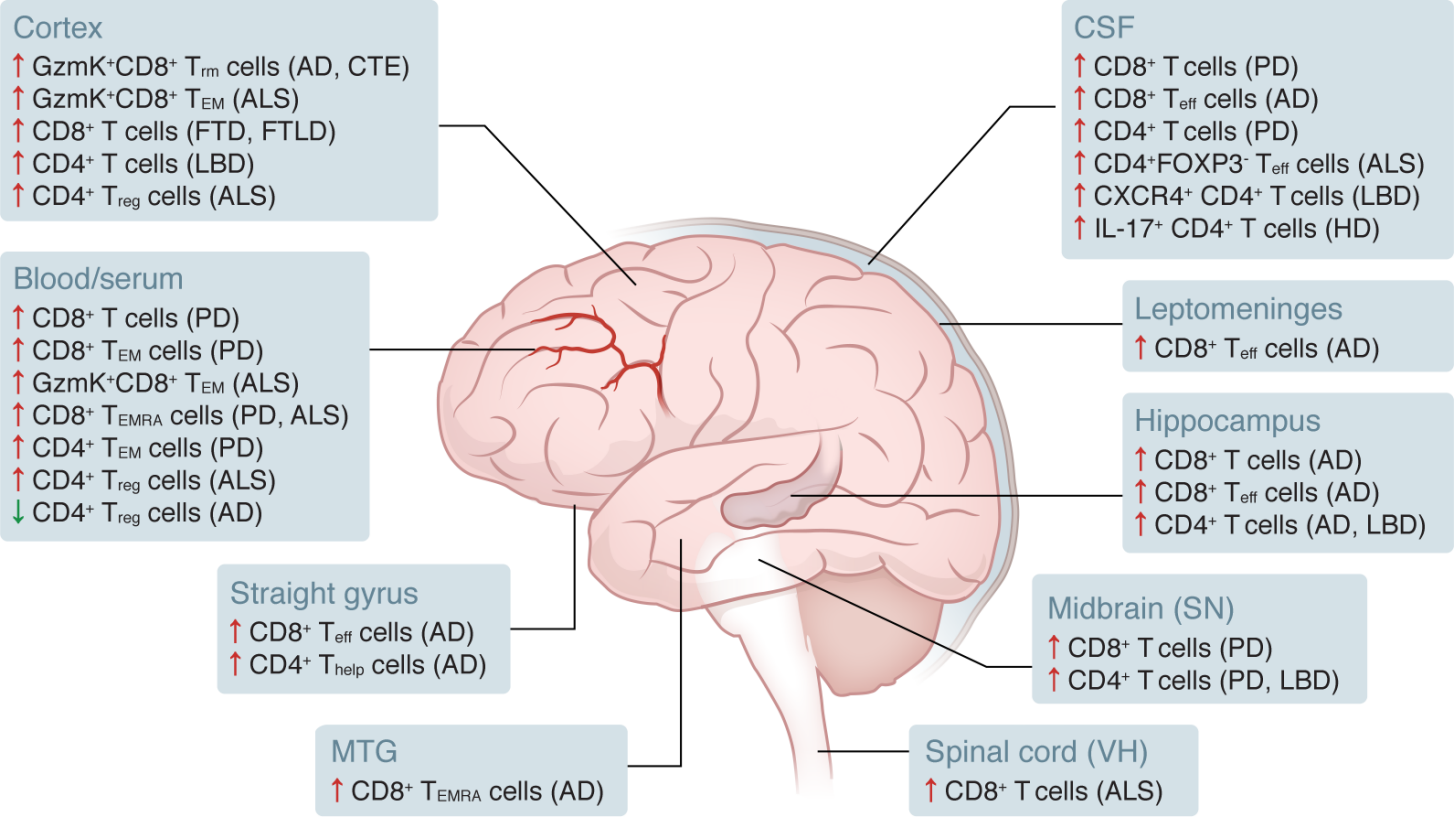
CNS distribution of CD4^+^ and CD8^+^ T cells across neurodegenerative diseases. Brain regions where CD8^+^ and CD4^+^ T cells have been detected in neurodegenerative diseases. MTG, middle temporal gyrus; SN, substantia nigra; VH, ventral horn; Thelp, helper; Teff, effector.

Adaptive immune involvement has emerged as a shared feature across several neurodegenerative diseases. CD4^+^ and CD8^+^ T cells were first observed in the brains of patients with AD in the 1980s (Figure 2) (139, 140). Ongoing work continues to define T cell subpopulations and clarify how the adaptive immune system contributes to neurodegenerative diseases. Overall, these early discoveries established T cells as important immune constituents within the degenerating CNS, prompting deeper investigation into their roles.

### CD4^+^ T cells.

Growing evidence indicates that CD4^+^ T cells exhibit diverse and disease-specific functions across multiple neurodegenerative disorders (Figure 2). In the CSF of patients with AD, the frequency of CD4^+^ T cells decreases over time, yet these cells show evidence of clonal expansion with reduced diversity (141). Transfusion of Aβ-restricted CD4^+^ T cells into APP/PS1 transgenic mice was neuroprotective and prevented cognitive decline while showing little infiltration into the CNS (142). Likewise, transfer of Aβ-specific CD4^+^ T cells polarized toward a T helper 2 (Th2) phenotype into APP/PS1 mice reversed cognitive decline and synaptic loss (143). CD4^+^ T cells are also recruited into brain parenchyma in ALS, PD, and LBD (144–148). In the SOD1 ALS model, loss of CD4^+^ T cells in RAG2 or TCRβ-deficient mice reduced survival and diminished inflammatory gene expression in brain and spinal cord parenchyma (149, 150). In contrast, α-syn–specific CD4^+^ T cells in PD models were neurotoxic and promoted inflammation in *Rag1*-knockout mice (144, 147). Overexpression of α-syn enhanced recruitment of IFN-γ–producing CD4^+^ T cells, and depletion of these cells reduced neuronal loss (151). In LBD, CXCR4-expressing CD4^+^ T cells and the CXCR4 ligand CXCL12 were increased in the CSF, and CXCL12 levels correlated with neuroaxonal damage (145). Collectively, these studies indicate that CD4^+^ T cells can be protective in some neurodegenerative contexts but pathogenic in others, particularly in the presence of α-syn.

The inconsistencies among diseases may reflect differences in CD4^+^ T cell subpopulations. Tregs, defined by expression of FOXP3, play essential roles in immune regulation (152). Total (CD25 high and CD127 low to negative) and resting (CD45RA^+^ and CD25 dim) Tregs were reduced in peripheral blood from patients with AD (153). In ALS, the ratio of Tregs to total CD4^+^ T cells inversely correlated with disease progression (146). Further analysis showed that high frequencies of CD4^+^FOXP3^neg^ effector T cells in blood and CSF predicted poor survival, whereas high levels of activated Tregs and a high ratio of activated-to-resting Tregs were associated with improved survival (154). In PD, increased CD45RO^+^FAS^+^ effector memory CD4^+^ T cells were observed along with dysfunctional Tregs (155). Patients with mutations in the huntingtin (*HTT*) gene showed reduced Treg frequencies and increased IL-17–expressing Th17 cells in CSF. Whether enrichment of beneficial CD4^+^ T cell subsets could slow disease remains an open question. In one study, PD-1 blockade increased the abundance of FOXP3^+^CD4^+^ Tregs, including PD-1^+^FOXP3^+^CD4^+^ subsets, in TE4 tauopathy mice, which was associated with reduced pTau accumulation and neurodegeneration (136). These observations together suggest that the balance between regulatory and effector CD4^+^ T cell subsets may critically shape neurodegenerative disease progression and could represent a therapeutic target.

### CD8^+^ T cells.

As interest in adaptive immunity in neurodegenerative disease has grown, CD8^+^ T cells have become increasingly recognized as important contributors to CNS pathology, but their disease context–dependent functions remain unclear (Figure 2). Extravascular CD3^+^ and CD8^+^ T cells were detected in the hippocampus of postmortem AD brains, and CD3^+^ T cell numbers correlated with tau pathology but not amyloid plaques (156, 157). Depletion of CD8^+^ T cells in APP/PS1 mice did not alter disease course, though it did change neuronal gene expression. Notably, depletion in this study was initiated at 12 months of age when marked pathology was already present (157). In PD, CD8^+^ T cell numbers increased and correlated with neuronal death, although it remains uncertain whether this is causative or reactive (158). α-syn–specific T cells emerge in the blood before motor symptoms in PD, peak soon after symptom onset, and decline as disease progresses. Reconstitution experiments in RAG-deficient mice demonstrated that both CD4^+^ and CD8^+^ T cells can independently induce neuronal loss in mice overexpressing human A53T α-syn through antigen-specific responses (144). CD8^+^ T cells were also present in patients with FTD with the P301L mutation and in the hippocampus of THY Tau22 mice. In that model, depleting CD4^+^ and CD8^+^ T cells with an anti-CD3 antibody rescued behavior defects despite no effect on pTau deposition (159). In TE4 tauopathy mice, microglia facilitated recruitment of T cells to the dentate gyrus, and depletion of microglia or CD4^+^ and CD8^+^ T cells reduced tau pathology and neurological decline (136). Overall, the evidence points to highly context-dependent roles for CD8^+^ T cells in neurodegenerative disease.

To understand these divergent effects, attention has shifted toward defining CD8^+^ T cell subsets and the mechanisms directing their localization and activity in the CNS. CD8^+^ tissue–resident memory (Trm) cells depend on CXCR6 for localization to peripheral tissues (160). In aged APP/PS1 mice, CXCR6^+^ CD8^+^ T cells were enriched in the hippocampus relative to blood, and their transcriptomic profile resembled antiviral and antitumor responses (161). In 5xFAD mice, neuroprotective CD8^+^ T cells accumulated in the brain and restricted Aβ plaque growth. Single-cell sequencing identified CXCL16 expression by microglia and CXCR6 expression by CD8^+^ T cells, and this signaling axis was required for their protective effect. Combined single-cell and TCR sequencing revealed clonally expanded CD4^+^ and CD8^+^ T cells that transitioned from activated to exhausted states, with the activated subset exhibiting neuroprotective properties (162). This axis appears conserved in humans, as cognitively impaired individuals showed elevated CXCR6^+^CD8^+^ T effector memory (Tem) cells in CSF. Immunostaining of AD brain tissue confirmed CXCR6 expression on CD3^+^ T cells and CXCL16 expression by Iba1^+^ microglia, supporting microglia-mediated recruitment into the parenchyma (163). Extending these findings to the leptomeningeal compartment, a recent study identified clonally expanded PD-1^+^ CD8^+^ Trm cells in the leptomeninges of patients with AD and ALS, with substantial overlap between brain and leptomeningeal TCR repertoires, while additional tissue-specific clones emerged in AD. In AD, the degree of CD8^+^ Trm clonal expansion correlated positively with microglial TGFB2 expression, raising the possibility of functional crosstalk between meningeal T cells and parenchymal immune cells (164). Together, these findings underscore the importance of Trm-like CD8^+^ T cell populations in shaping immune dynamics during neurodegeneration, while raising important questions regarding how distinct memory CD8^+^ T cell subsets contribute to disease progression.

Additional memory CD8^+^ T cell subsets with strong cytotoxic potential include terminally differentiated effector memory (Temra) cells (165). Temra cells accumulated in the CSF during mild cognitive impairment, an early stage of AD (166). In another study, increased numbers of CD8^+^ Temra and Tem cells in the hippocampus and leptomeninges correlated with cognitive decline in AD. Temra cells also clonally expand in the CSF of patients with AD, with some clones recognizing EBV, although this does not imply a causal relationship between EBV exposure and AD (167). A sex-specific increase in CD8^+^ Tem and Temra cells has also been reported in females with idiopathic PD who exhibit reduced CD8^+^ Tregs and an elevated Temra-to-Treg ratio (168). In ALS4, caused by mutations in senataxin (*SETX*), patients show clonally expanded CD8^+^ Temra cells in circulation and CD8^+^ T cells in the ventral horn of the spinal cord. Mice carrying the L398S *Setx* mutation similarly display expanded PD-1^+^CD8^+^ Temra cells across spinal cord, brain, and blood, indicating that antigen-specific CD8^+^ T cell responses contribute to disease development (169). These findings highlight the diverse ways in which memory CD8^+^ T cell subsets become activated and expanded across neurodegenerative diseases, paving the way for the identification of additional populations with specialized functions.

As research tools advance, T cell subsets continue to be defined with increasing precision. A recently described population of granzyme K–expressing (GzmK-expressing) CD8^+^ T cells has been implicated in chronic tissue inflammation and complement activation (170–174). Although less cytolytic than other granzymes, GzmK promotes antiviral responses through inflammatory mechanisms (173, 175). GzmK^+^ CD8^+^ T cells were identified in the CSF of patients with ALS, in post mortem brains of individuals with ALS, AD, chronic traumatic encephalopathy (CTE), and advanced age, and in the spinal cords of P301S tauopathy mice (137, 176, 177). In sporadic ALS, these cells clonally expanded in CNS and blood and were enriched in parenchyma and perivascular regions compared with controls (177). In the P301S model, microglia upregulated CD11c and MHCII, supporting pTau clearance. Under conditions of sustained inflammation, clonally expanded PD-1^+^CD103^+^CD8^+^ Trm cells with a noneffector transcriptional profile infiltrated the spinal cord parenchyma, interacted with maladaptive microglia, and deposited extracellular GzmK. CD8 deficiency exacerbated pTau accumulation and produced a distressed microglial population containing neuronal transcripts, leading to accelerated neurological decline (137). These findings suggest that GzmK^+^CD8^+^ T cells are protective in this tauopathy model. By contrast, in 3xTg AD mice, CD103^–^ CD8^+^ Trm cells expressed high levels of GzmK and adopted a gene signature associated with neurodegeneration. Depletion of peripheral CD8^+^ T cells reduced Aβ and pTau accumulation, improving cognition. Coculture experiments confirmed that these CD103^–^CD8^+^ Trm cells were directly neurotoxic through GzmK-mediated engagement of protease activation receptor 1 (176). Further work is needed to determine the disease specificity of GzmK^+^CD8^+^ T cell functions and whether these cells can be therapeutically targeted.

Overall, CD8^+^ T cells display diverse roles in neurodegeneration, shaped by tissue residency, antigen specificity, and interactions with microglia. Across diseases, these cells infiltrate affected CNS regions, clonally expand, and adopt diverse transcriptional states ranging from protective to pathogenic, as illustrated by CXCR6^+^ Trm-like cells, cytotoxic Tem/Temra subsets, and GzmK^+^ populations. These observations highlight the need to define the drivers of CD8^+^ T cell activation, the antigens that shape their clonal repertoires, and the microenvironmental factors that determine whether they support repair or promote neurodegeneration. Integrating refined T cell subset classification with mechanistic studies of T cell–microglia interactions will be crucial for developing precise immunomodulatory therapies tailored to specific neurodegenerative contexts.

## Factors shaping neurodegenerative immune responses

**Figure 3:**
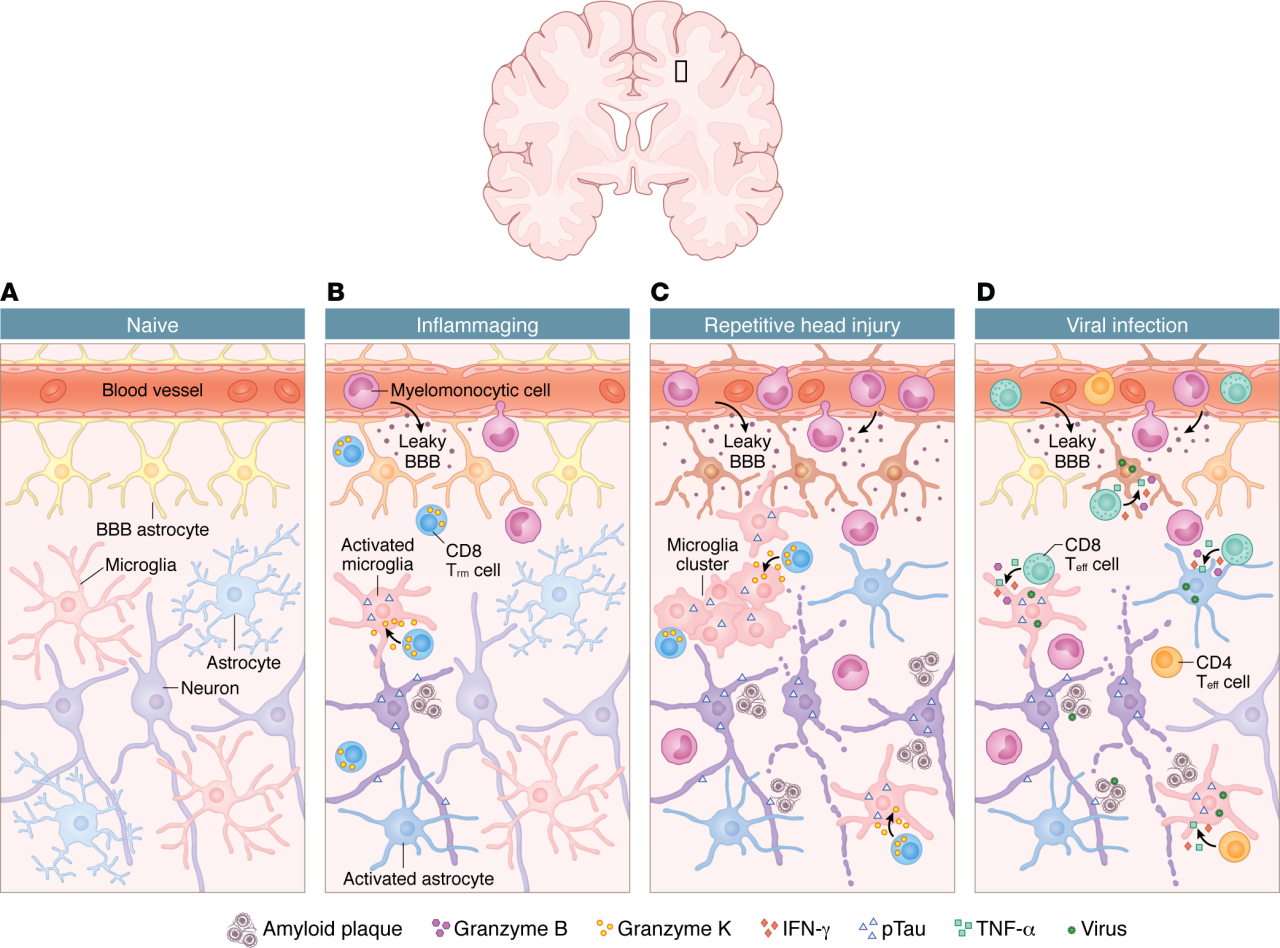
External factors that reshape CNS immunity and influence neurodegenerative risk. External factors influence both the initiation and progression of neurodegenerative disease by activating distinct immune pathways. The illustration depicts 4 conditions that alter CNS immunity. (**A**) In the naive state, intact vasculature, BBB-associated astrocytes, and homeostatic glia maintain neural function. (**B**) Inflammaging is characterized by BBB decline, infiltration of peripheral immune cells, including granzyme K–expressing CD8^+^ T cells, and accumulation of circulating material such as fibrinogen, albumin, and ions that drive chronic low-grade inflammation and aberrant protein deposition. (**C**) Repetitive head injury induces persistent BBB disruption, reduced vascular integrity, and reactive astrocytes and microglia that cluster around accumulating Aβ and pTau; CD8^+^ T cells deposit granzyme K onto these microglial aggregates. (**D**) During viral infection, microglia and astrocytes respond to pathogen-associated signals and recruit myelomonocytic cells along with CD4^+^ and CD8^+^effector T cells, which utilize IFN-γ, TNF-α, and granzyme A/B/C to control infection.

Although our understanding of immunopathogenesis in neurodegenerative disease continues to expand, these disorders remain highly complex, with multifactorial and often unclear etiologies. Insights into the roles of resident and infiltrating immune cells highlight the need to consider additional influences that shape how immune responses affect neuronal health and overall disease risk. Examining aging, repetitive injury, and viral infection provides a framework for understanding how diverse biological and environmental pressures modulate CNS immunity during neurodegeneration (Figure 3).

### Inflammaging.

Aging is one of the primary risk factors for neurodegenerative diseases such as AD and PD. In youth, homeostatic microglia survey the CNS, astrocytes maintain the BBB, and only limited numbers of T cells enter the parenchyma. With advanced age, however, chronic low-grade inflammation develops in a process termed inflammaging (Figure 3B), during which many of the immune pathways described above become dysregulated (178). For example, plasma mtDNA levels increase after age 50 and correlate with elevated proinflammatory cytokines such as TNF-α and IL-6 (179). Consistently, activation of cGAS in aged mice produced cognitive impairment, neuronal loss, and neuroinflammation similar to that seen in neurodegenerative models (180). Systemic inflammation increases with age, and these immune alterations are accompanied by profound structural and functional changes within the CNS that further heighten vulnerability to neurodegenerative pathology.

Aging also compromises the integrity of the BBB. Increased permeability promotes leukocyte extravasation into the parenchyma (181) and this process is exacerbated by age-related declines in microvasculature density (182, 183). As a result, cytotoxic CD8^+^ T cells can accumulate and damage neurons through granzyme B activity (184). In humans, a distinct exhausted-like subset of granzyme K-expressing CD8^+^ T cells expands with age, and aged mice exhibit a PD1^+^GzmK^+^CD8^+^ population that mirrors the phenotype seen in tauopathy models (137, 172, 185). Together, these findings suggest that age-related immune changes can both impair CNS homeostasis and predispose individuals to neurodegenerative disease development.

### Repetitive head injury.

External insults such as repetitive head injury can further disrupt CNS immune homeostasis and accelerate neurodegenerative processes (Figure 3C). Traumatic brain injury (TBI) is strongly linked to later development of neurodegenerative diseases including AD, PD, and CTE (186–188), even when injuries occur during childhood (189). Following mild TBI, distressed cells release DAMPs such as mtDNA and HMGB1 (190), triggering microglial activation. Microglia clear debris and reinforce weakened regions of the BBB and glia limitans superficialis, which becomes vulnerable following astrocyte death (191–194). However, repeated injuries can cause these barrier-supporting microglia to become reactive and die, leaving openings that allow entry of myelomonocytic cells (195, 196). If the CNS cannot restore homeostasis after injury, risk of CTE increases. CTE is characterized by pTau accumulation and widespread glial activation. Postmortem CTE brains often show additional proteinopathies including TDP-43 inclusions, Aβ plaques, and α-syn deposits (197–200). Across experimental TBI paradigms, injury precipitates acute and chronic proteinopathy, including elevations in total and oligomeric tau (201), transient increases in soluble and insoluble Aβ species (202), enhanced hippocampal Aβ deposition at later time points (203, 204), and broader neurodegenerative protein signatures following repetitive injury (205). Because CTE can only be diagnosed histologically, early detection after TBI is a critical unmet need. Understanding how repeated injuries promote chronic immune activation may reveal mechanisms shared with other neurodegenerative diseases and highlight intervention points independent of age or genetic risk.

### CNS viral infection.

Like repetitive injury, viral infections can profoundly alter CNS immunity and shape susceptibility to neurodegeneration (Figure 3D). Many immune pathways implicated in neurodegeneration originally evolved to combat invading pathogens, and recent studies link both neurotropic and nonneurotropic viral infections to increased risk for, or accelerated progression of, neurodegenerative diseases (206–210). Viruses access the CNS through several mechanisms, including infection of endothelial cells or astrocytes (211), or through inflammation-induced barrier disruption similar to that seen in aging and TBI (212, 213). Once present, viral products activate microglia and infiltrating myeloid cells through both PAMP- and DAMP-dependent pathways, promoting secretion of IFN-I and other inflammatory mediators involved in viral clearance (214). Consistent with a potential role for herpesviruses in dementia risk, natural experiment–based studies have reported that herpes zoster vaccination (both live-attenuated and recombinant) is associated with reduced incidence of dementia and prolonged dementia diagnosis–free survival (215–217). Although causality and mechanisms remain to be established, these findings suggest that antiviral vaccination or immune modulation may influence dementia risk and disease trajectory.

As the antiviral response progresses, these innate signaling events rapidly recruit adaptive immune cells into the CNS. During early infection, CD4^+^ and CD8^+^ T cells traffic into the CNS, with CD8^+^ T cells comprising the majority of infiltrating lymphocytes and relying on IFN-γ, TNF-α, and perforin for antiviral effector activity (218–220). Repeated peripheral viral infections have been shown to expand and diversify the pool of CD8^+^ Trm cells in the CNS, thereby enhancing protection against subsequent infections (221, 222). However, persistence of CD8^+^ T cells after viral clearance can promote chronic microglial activation, synaptic loss, and cognitive deficits (223). Viral products may also interact with pathogenic proteins implicated in neurodegenerative diseases. HSV-1 DNA colocalized with Aβ plaques in both AD and control brains, although unbound HSV-1 DNA was more prevalent in controls, suggesting more efficient viral clearance under normal conditions (224). Aβ has been proposed to function as an antimicrobial peptide and may be upregulated as part of a protective innate response. In 5xFAD mice infected with HSV-1, Aβ plaque formation accelerated and survival improved (225), although other studies found no protective effect across HSV-1 strains (226). These findings support a model in which viral infections, even after resolution, reshape the CNS immune environment in ways that may increase susceptibility to future neurodegenerative processes, while also raising the possibility that certain infections or antiviral responses could transiently modulate pathology in protective or compensatory ways.

## Concluding remarks

The CNS parenchyma can mount immune responses, yet these responses are carefully regulated to preserve neural function. Microglial engagement of self-derived DAMPs, together with the recruitment of diverse T cell populations, illustrates how multiple immune compartments respond dynamically to pathogenic protein accumulation. Advances such as single-cell sequencing have expanded our understanding of these processes by revealing distinct immune subsets and clarifying how innate and adaptive pathways intersect. Together, these insights establish immune dysregulation as a unifying theme across the spectrum of neurodegenerative disorders.

Neurodegenerative diseases arise from a complex interplay of biological and environmental factors that shape not only the timing and magnitude of immune activation but also the programming of microglia, T cells, and other leukocytes. Aging, repetitive head injury, and viral infection each restructure the CNS immune environment by promoting chronic inflammation, altering microglial and T cell phenotypes, and shifting the balance between protective and damaging responses. Many of the immune pathways involved show context-specific and stage-dependent functions. Microglial activation may aid early clearance of aberrant proteins but become maladaptive with prolonged stimulation, and CD8^+^ T cells can either support tissue repair or promote neuronal injury depending on antigen specificity, effector programming, and local signaling cues. Appreciating both the temporal dynamics and cell-intrinsic states of immune populations is essential for interpreting immune signatures and for identifying when immunomodulatory intervention is most likely to be beneficial.

These discoveries have strengthened the rationale for developing targeted immunotherapies to slow or prevent neurodegenerative disease progression. Approaches under investigation include modulation of microglial receptors such as TREM2, inhibition of pathways such as cGAS and STING, and strategies that reshape T cell activity through immune checkpoint molecules like PD-1 or by enhancing regulatory T cell function. In the future, adoptive immunotherapies that involve transferring expanded or engineered T cell subsets with neuroprotective properties may offer new opportunities to reinforce beneficial immune programs or counter harmful ones. Continued integration of human and animal studies, high dimensional profiling, and longitudinal sampling will be vital for determining causality, identifying therapeutic windows, and distinguishing adaptive from maladaptive immune remodeling. A deeper understanding of how immune signaling networks interact with genetic, environmental, and age-related stressors will be essential for guiding the development of effective immunomodulatory therapies.

## Conflict of interest

The authors have declared that no conflict of interest exists.

## Funding support

This work is the result of NIH funding, in whole or in part, and is subject to the NIH Public Access Policy. Through acceptance of this federal funding, the NIH has been given a right to make the work publicly available in PubMed Central. The contributions of the NIH authors are considered Works of the United States Government. The findings and conclusions presented in this paper are those of the authors and do not necessarily reflect the views of the NIH or the U.S. Department of Health and Human Services.

- The Defense Health Agency (CA#HU00012520039/HJF#10002).
- The Intramural Research Program of the National Institute of Neurological Disorders & Stroke (NINDS) within the National Institutes of Health (NIH).

## Version 1 04/15/2026

Electronic publication
